# The use of solid phase microextraction for metabolomic analysis of non-small cell lung carcinoma cell line (A549) after administration of combretastatin A4

**DOI:** 10.1038/s41598-018-36481-2

**Published:** 2019-01-23

**Authors:** Karol Jaroch, Ezel Boyaci, Janusz Pawliszyn, Barbara Bojko

**Affiliations:** 1 Department of Pharmacodynamics and Molecular Pharmacology, Faculty of Pharmacy, Collegium Medicum in Bydgoszcz, Nicolaus Copernicus University in Toruń, Toruń, Poland; 2 Department of Chemistry, Middle East Technical University, Ankara, 06800 Turkey; 3 Department of Chemistry, University of Waterloo, 200 University Avenue West, Waterloo, ON Canada

## Abstract

Use of solid phase microextraction (SPME) for cell culture metabolomic analysis allows for the attainment of more sophisticated data from *in vitro* cell cultures. Moreover, considering that SPME allows the implementation of multiple extractions from the same sample due to its non/low-depletive nature, time course studies using the same set of samples are thus facilitated via this method. Such an approach results in a reduction in the number of samples needed for analysis thus eliminates inter-batch variability related to biological variation occurring during cell culturing. The current work aims to demonstrate the capability of SPME for measurements of combretastatin A4 (CA4) effectiveness on non-small cell cancer cell line. A cultivation protocol was established in the 96-well plate, and a fiber format of SPME was selected for metabolite extraction. The extracellular metabolic pattern of cells was changed after administration of the tested drug. This suggests pharmacological activity of the administered compound towards the studied cell line model. Results support that the use of direct immersion SPME for analysis of cell cultures does not affect cells growth or contaminate sample. Consequently, SPME allows the attainment of accurate information regarding drug uptake, metabolism, and metabolomic changes in the studied cells induced by exposure to the drug simultaneously in a single experiment.

## Introduction

Nowadays, most standard anticancer therapies incorporate the use of cytotoxic agents. Due to their lack of selectivity to cancer tissue, their administration often causes wide-ranging side effects^1^. One such modern anticancer therapy strategy entails the inhibition of neovascularization in tumor tissue; accordingly, drugs that act via this mechanism are classified as vascular disrupting agents (VDAs). The efficiency of VDAs is attributed the fact that most cancer tissues of a diameter larger than 1–2 mm require new blood vessels for further development^2,3^. Thus, the lack of oxygen and nutrients caused by insufficient blood flow in these tissues contributes to the death of tumor cells^4^.

**Figure 1:**
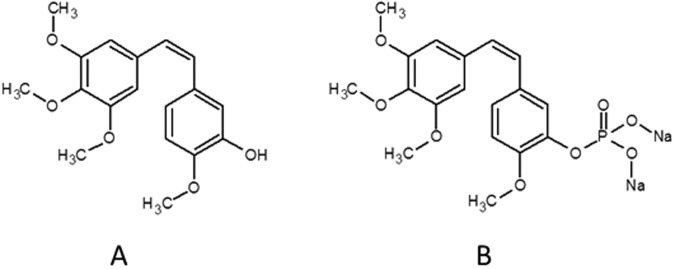
Chemical structures of combretastatin A4 (CA4, **A**) and combretastatin A4 phosphate disodium salt (CA4P, **B**).

Members of the combretastatin family are known to cause vascular disruption to different degrees. Their mechanism of action is based on tubulin polymerization inhibition, which leads to changes in the endothelial cell cytoskeleton, resulting in the malformation of endothelial cells. The loss of integrity and higher permeability of blood vessels that occurs due to these malformations, in turn, leads to the clogging of said blood vessels, and the eventual death of the cancer tissue surrounding them^5^. Among combretastatins, combretastatin A4 (CA4, Fig. 1A.) is regarded as the most potent naturally occurring combretastatin. Its prodrug, combretastatin A4 phosphate disodium salt (CA4P, Fig. 1B.), is currently being tested in various clinical trials^6^, including patients suffering from non-small cell lung cancer^7,8^. While the most important clinical feature afforded by CA4 relates to its role in the collapse of blood vessels of cancer tissue, CA4 has also been shown to cause cell death of cancer cells, either directly via apoptosis, or indirectly through tumor autophagy and necrosis^9^. That is why the biological effect of combretastatins can be established not only on blood vessel endothelial cell but also directly on cancer cell lines, as done in current work.

As a useful tool in oncology research, the non-small cell lung cancer (NSCLC) adenocarcinoma cell line A549 cell line has been established to provide an adequate representation of alveolar type II pulmonary epithelial cells of human lung^10^. Since its introduction in 1972, this cell line has been widely used in toxicological screenings of new drug candidates. In addition, this cell line is listed in the National Cancer Institute (NCI) cancer panel NCI-60, which lists cell lines suitable for screening of new drug candidates^11^. Cell lines represent a valuable tool in preclinical studies, as they enable the development of preclinical models, allowing researchers to test the efficacy of a developed therapeutic strategy prior to human trials. Within this context, it is anticipated that employment of *in vitro* “-omics” strategies to elucidate mechanisms of action and assess drug effectiveness will continue to expand in the field of oncology, as these assays enable very deep insights regarding given biological matrix. For instance, metabolomics studies can reveal vital information regarding small molecules within a biological sample, thus enabling a better understanding of the particular biochemistry of a given matrix, such as that of cancer cells. In this respect, solid phase microextraction (SPME) is a valuable platform for metabolomics studies, having been utilized for investigations pertaining to a wide variety of biological matrices such as fruits, leaves, animal tissues (both *in* and *ex vivo*), and biofluids (serum, plasma, whole blood, urine saliva), among others^12^. Solid phase microextraction was first introduced in 1990 as an alternative, greener microextraction technique^13^. As an equilibrium-based solvent-less technique, biocompatible SPME sorbents used for LC-based applications extract only low molecular weight compounds from a given sample into the coating (sorbent), providing good sample cleanup and selectivity. The principle of extraction lies in the equilibrium state established between the concentrations of target analyte(s) in the sample and fiber coating. An important feature of SPME lies in its ability to offer non-exhaustive extraction under certain conditions. Due to this capability, the homeostasis of drug-protein binding will not be disturbed during the procedure, given that suitable parameters are selected for this purpose. Furthermore, the extraction step sufficiently inhibits enzymatic conversion of extracted moieties by preventing adhesion of macromolecules, including enzymes, to the sorbent and thus leads to immediate metabolism quenching. Described quenching occurs simultaneously with the partition of analytes into the sorbent. Such effective separation in the “native sample” enables to preserve species, which enzymatic conversion is faster than quenching procedure performed on the collected sample e.g. snap freezing. This is particularly visible when *in vivo* vs. *ex vivo* analyses are compared^14^. Given the above described features, SPME presents itself as a very suitable tool for metabolomics analysis. Thanks to the excellent clean-up afforded by the SPME procedure, extracts can be directly injected to LC-MS, which nowadays is a gold standard in metabolomics analysis. Further details regarding the specific characteristics of SPME can be found elsewhere^15,16^.

Headspace SPME (HS-SPME), whereupon a sorbent is exposed to the headspace of a sample, has been widely used for metabolomics studies of cell cultures. To date, the A549 cell line has only been sampled via HS-SPME for analysis of volatile organic compounds (VOCs)^17–20^. In the current study, direct immersion SPME is used for the first time for analysis of the metabolome of cancer cells during exposure to the chemotherapeutic agent combretastatin A4 phosphate disodium salt. The main goal of the presented study was to show the capability of SPME in the evaluation of metabolomic changes after administration of combretastatin A4 or combretastatin A4 phosphate. Moreover, as we employed two different setups of SPME, the flexibility of the extraction method was also shown as both SPME protocols enable performing other assays on cells.

## Materials and Methods

### Materials

A549 cell line was cultivated at 37 °C and 5% CO_2_ with Dulbecco’s Modified Eagle Medium (DMEM with 4.5 g L^−1^ glucose, L-glutamine and sodium pyruvate, Corning, Oneonta, USA), supplemented with 10% Fetal Bovine Serum (FBS, Biowest, Nuaillé, France), as well as antibiotics and antimycotics (Sigma-Aldrich, Poznań, Poland). Octadecyl silica (C18) with benzenesulfonic acid SPME fibers consisted of an 8 mm coating length (Supelco, Bellefonte, USA) were utilized for the first experiment (see protocol 1 below). SPME fibers utilized for direct immersion using the 96-well plate were manufactured in house, as described in^21^. Fibers were manufactured in-house with 5 μm hydrophilic−lipophilic balanced (HLB) particles provided by Waters (Wilmslow, U.K.), and were consisted of a coating length of 2 mm (see protocol 2 below).

### LC-MS analysis

In order to obtain more comprehensive data and cover a wide range of compounds two separate chromatographic methods were used. Chromatographic separations were performed using reversed phase and HILIC columns: pentafluorophenyl (PFP) Discovery HS F5, 100 mm × 2.1 mm, 3 μm, (Supelco, Bellefonte, PA, USA); mobile phase: A: 99.9% water +0.1% formic acid B: 99.9% acetonitrile +0.1% formic acid, and Luna HILIC, 100 mm × 2.0 mm, 3 μm, 200 A, (Phenomenex, Torrance, CA, USA); mobile phase: A: acetonitrile/ammonium acetate buffer (9:1 v/v, 20 mM effective salt concentration) B: acetonitrile/ammonium acetate buffer (1:1 v/v, 20 mM effective salt concentration). The LC gradient for the PFP column was 0–3 min 0% B, 3–25 min linear gradient to 90% B, 25–34 min 90% B, 34–40 min 0% B, and a flow of 0.3 mL × min^−1^. The HILIC column gradient was set as follows: 0–3 min 0% B, 3–8 min linear gradient to 100% B, 8–12 min 100% B, 12–20 min 0% B, and a flow of 0.4 mL × min^−1^. Both gradients were adopted from previous work of Vucovic *et al*.^22^. All solvents utilized for SPME and LC-MS analysis were LC-MS grade (Sigma-Aldrich, Poznań, Poland).

The LC-MS system consisted of a Dionex UltiMate 3000 RS autosampler, Dionex Ultimate 3000 RS pump (Thermo Fisher Scientific, Dionex, Germany), and a Q-Exactive Focus high resolution mass spectrometer (Thermo Fisher Scientific, Germany). The MS was operated in both positive and negative ionization modes. MS in positive ionization mode was run with HESI ion source parameters set as follows: spray voltage 1500 V, capillary temperature 300 °C, sheath gas 40 a.u., aux gas flow rate 15 a.u., aux gas flow rate 21 a.u., probe heater temperature 300 °C, S-Lens RF level 55%. For negative ionization mode, HESI ion source parameters were as follows: spray voltage 2500 V, capillary temperature 250 °C, sheath gas 47,5 a.u., aux gas flow rate 11 a.u., probe heater temperature 412 °C, S-Lens RF level 55%. Data acquisition was performed by Xcalibur software v. 4.0

### Cell culturing and extraction protocols

SPME extractions were carried out as part of two distinct investigations. In the first experiment, the A549 cell line was exposed to CA4 at an IC50 concentration level, which is designated as the concentration of CA4 needed to only inhibit cell growth by 50%. The SPME procedure was then carried out after three days of cultivation (Protocol 1). In the second experiment, cells were exposed to a high concentration of CA4P for three subsequent days prior to SPME sampling (Protocol 2). Protocol details are described below.

#### Protocol 1

**Figure 2:**
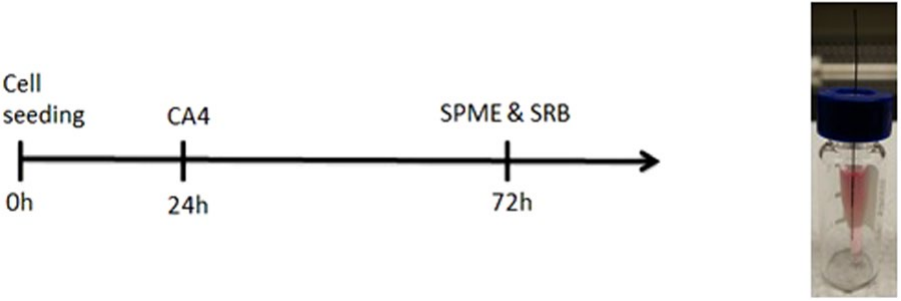
Left panel scheme of combretastatin A4 phosphate administration to A549 cell line, according to protocol 1. Right panel real view of sampling procedure in vial, according to protocol 2. (CA4-administration of combretastatin A4, SPME-solid phase microextraction, SRB- sulforhodamine B cytotoxicity assay).

Cells were first seeded onto a 96-well plate at two concentrations: 2000 and 1000 cell inoculum. After 24 h, CA4 was administrated by changing the cell media to new media containing 0.01 µM of the drug, which is the concentration of CA4 established to inhibit cell proliferation by 50% (data not shown). Cells were then left to grow for another 72 h at 37 °C and 5% CO_2_ (Fig. 2). Subsequently, 100 μL of cell media was collected from each sample into individual glass vial’s inserts, and SPME sampling, using 8 mm mix-mode fibers, was performed in direct infusion manner. Preconditioning of SPME fibers was carried out on a methanol:water (1:1, v/v) solution for 30 min, with orbital shaker agitation set at 1200 RPM. Fibers were then rinsed with water for 10 s without agitation. Extractions were carried out for 90 min with orbital shaker agitation at 1200 RPM from sample volumes of 100 μl. Fibers were then rinsed again with water for 10 s without agitation to remove any matrix components loosely attached to the fibers. Desorption was carried out in acetonitrile:water (4:1, v/v) for 90 min with orbital shaker agitation set at 1200 RPM, using a desorption solution volume of 100 μl. Subsequently, extracts were analysed with LC-MS system.

#### Protocol 2

**Figure 3:**
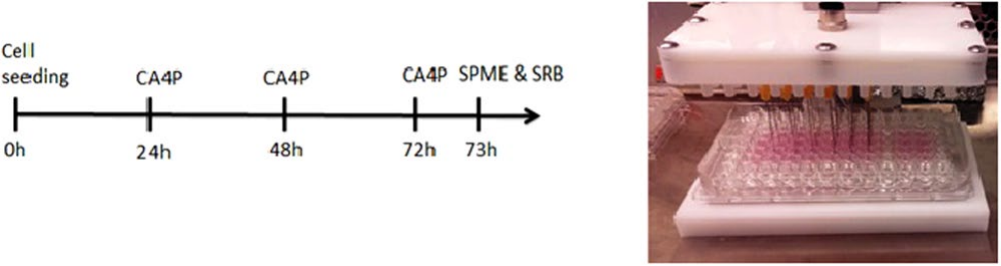
Left panel scheme of combretastatin A4 phosphate administration to A549 cell line, according to protocol 2. Right panel real view of 96-well plate sampling procedure, according to protocol 2. (CA4P-administration of combretastatin A4 phosphate, SPME- solid phase microextraction, SRB- sulforhodamine B cytotoxicity assay).

Cells were seeded onto a 96-well plate at concentration of 2000 cell inoculum. Administration of CA4P was performed 24, 48 or 72 hours after cell seeding as shown in Fig. 3. For each time point new triplicate of the samples was used. Administration of CA4P was carried out by replacing cell media with new media containing 100 µM of CA4P. SPME extraction, using in-house manufactured fibers, was performed directly from 96-well plate with all samples simultaneously analysed, therefore it may be considered as high throughput performance.

The extraction protocol was as follows: preconditioning of fibers was carried out in a methanol:water (1:1, v/v) solution for 30 min with orbital shaker agitation set at 1200 RPM. Following, fibers were rinsed with water for 10 s with no agitation. Extractions from matrix were carried out via direct immersion from 96-well plate samples. Sample volume was 100 μl, and extractions were carried out for 30 min, without agitation. Following extraction, fibers were rinsed with water for 10 s without agitation to remove loosely attached matrix constituents. Desorption was carried out in acetonitrile:water (4:1) for 90 min with orbital shaker agitation set at 1200 RPM, using a desorption solution volume of 100 μl. Subsequently, extracts were subjected to untargeted analysis on a high resolution mass spectrometer coupled to high performance liquid chromatography. Following SPME procedure, measurements of cytotoxicity based on the sulforhodamine B assay (SRB)^23^ were carried out on the investigated cell samples as the used extraction method does not consume biological material and it can be used for further analysis.

### Data processing and statistical analysis

For data processing and statistical analysis Compound Discoverer 2.1. (Thermo Fisher Scientific) software was used. The selected mass tolerance window was set up to 3 ppm, the signal-to-noise threshold to 3 and the max sample-to-blank ratio >5. Data was subjected to autoscaling and QC-based area was used for correction (min 80% coverage, max 30% RSD in QC, normalization by constant mean, fold change >2 and P < 0.05).

## Results and Discussion

**Figure 4:**
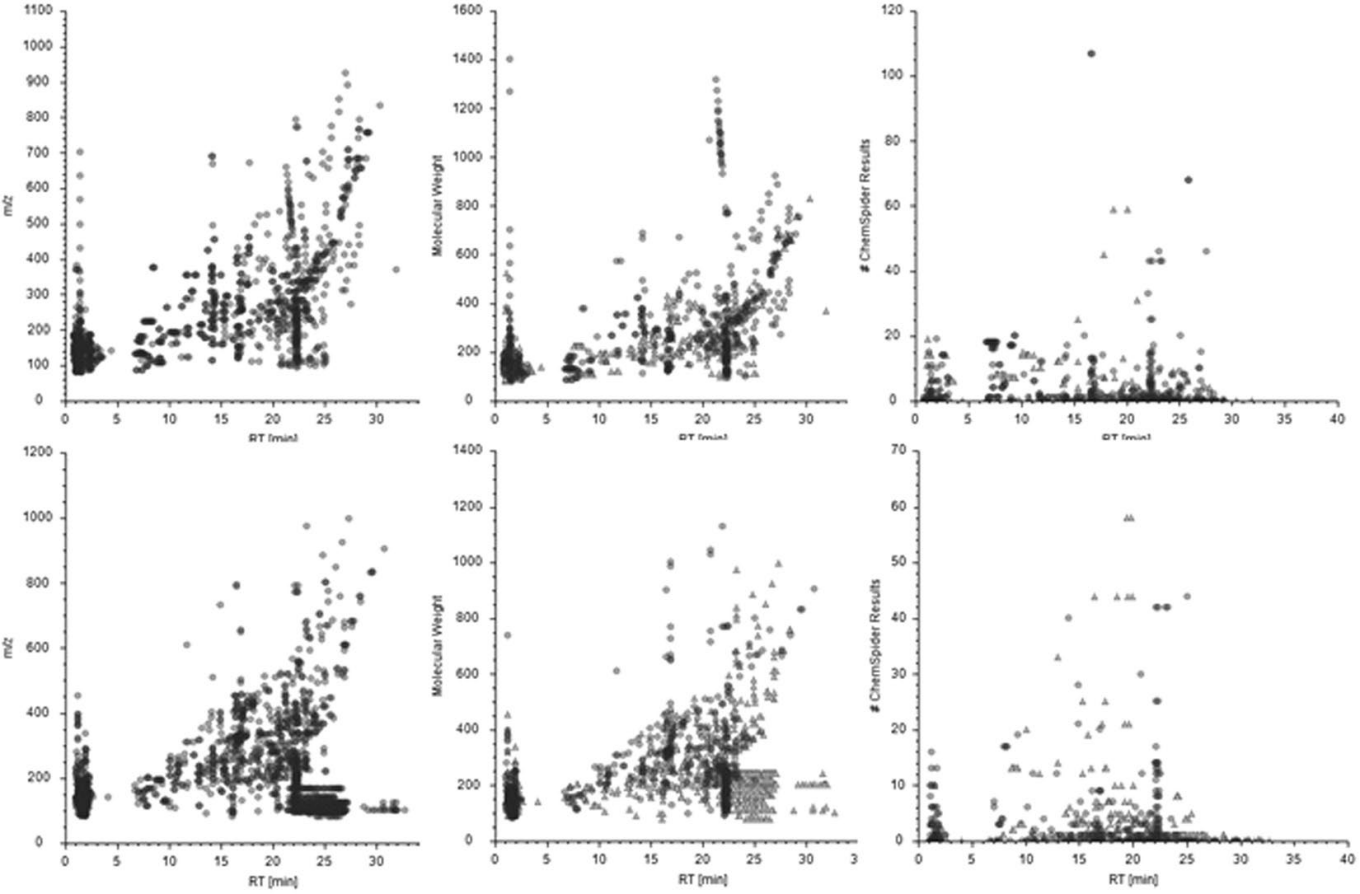
Charts representing number of features (left), annotated compounds (middle) and molecules identified in ChemSpider database (right). Top panel represents data from protocol number 1, bottom panel represents data from protocol number 2. m/z-mass to charge ratio.

The performance of the SPME probes used in the two protocols is shown in Fig. 4. The figure illustrates a comparison for the detected compounds, annotated molecules and metabolites identified based on the data collected in ChemSpider database. The data obtained for positive ionization mode and reversed phase separation was selected as representative results. The total number of features extracted by 8 mm mix-mode SPME fiber (used in protocol number 1) was 13466 (Fig. 4. top left). They were annotated into 617 compounds (Fig. 4, top middle) among which 319 were found in ChemSpider database (Fig. 4, top right). In the second experimental protocol, in which 2 mm HLB SPME fibers were employed, the total number of features was 11934 (Fig. 4. bottom left), of which 646 were annotated (Fig. 4 bottom middle) and 252 were putatively identified in ChemSpider database (Fig. 4. bottom right). Similar trends were found in other LC-MS configurations described in Materials and Methods section (data not shown). The chemistry of both coating types used in the study ensures broad analyte coverage, what is reflected by high number of molecular features observed in both protocols. To have head-to-head comparison preparation of the SPME probes of the same coating length and thickness (i.e. sorbent volume), similar particle size should be used. Also, different drug dosing might result in different metabolome changes. However, such comparison was beyond the scope of this study, which main goal was to demonstrate the applicability of SPME technology to cell line studies in different experimental setups. It was presented that SPME probes of longer coating might be successfully used for experiments conducted in Eppendorf tubes, vials or similar containers, while 2 mm coating can be completely immersed in a liquid with small sample volume, as in this case 100 μl of medium in 96-well plate. Moreover, employment of 2 mm SPME fibers give opportunity of performing multiple extractions from the very same cell culture medium, directly in 96-well plate, without need of medium change during each extraction. This feature is important in cell based *in vitro* studies because each medium change affects cell growth and proliferation rate which, in turn, may lead to biased conclusions.

**Figure 5:**
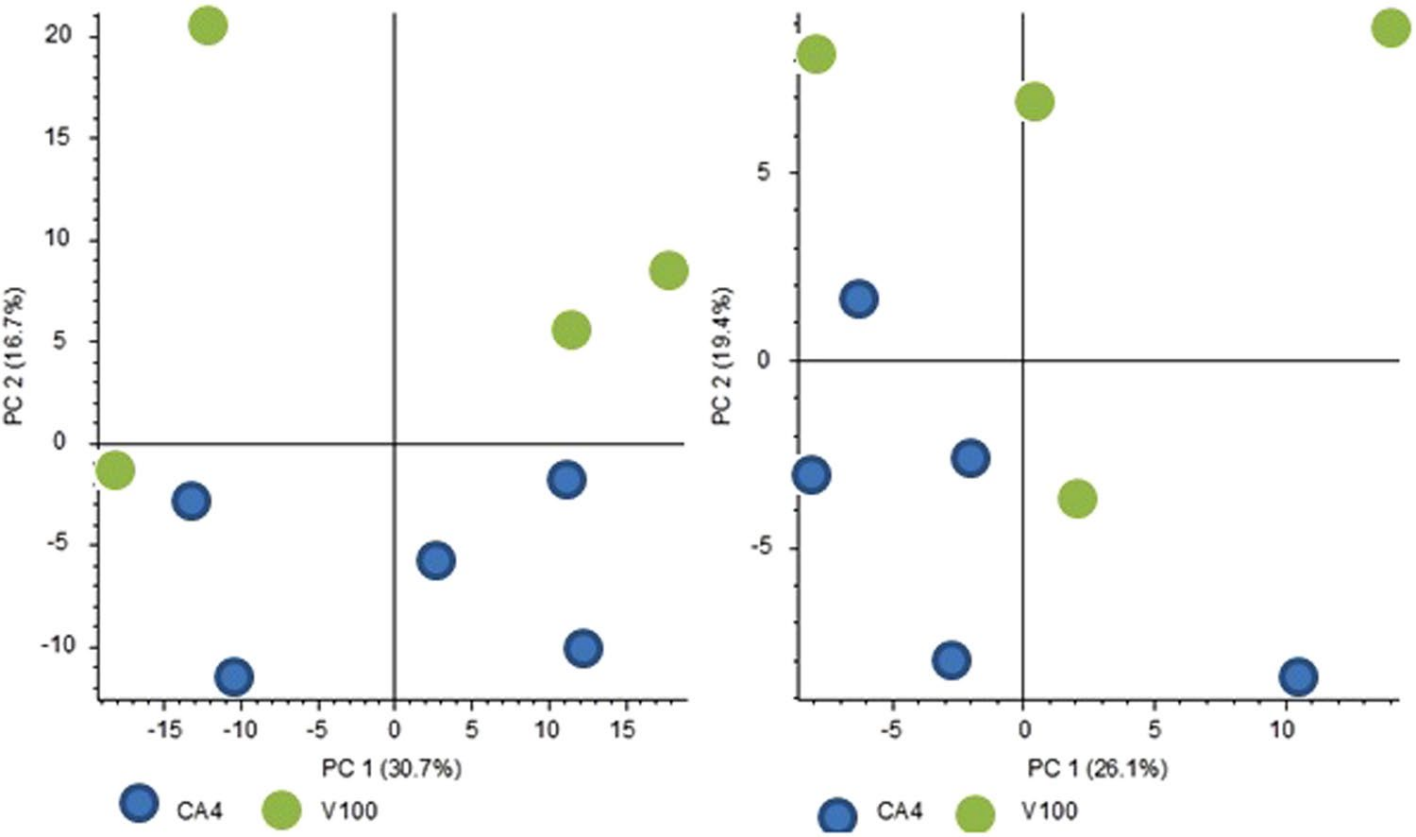
Principal Component Analysis diagram showing differences between cell treated with CA4 and vehiculum control. Left panel represents PFP column, right HILIC column both with positive ionization mode.

As can be seen from Fig. 5, the proposed protocol number 1 allows for discernment between A549 cell samples treated with CA4 and the vehiculum control. The observed differences between the studied system and its control are driven by the metabolites separated on both columns. Succinctly, the attained results indicate the involvement of metabolites of a wide polarity range in biochemical changes occurring in the studied system. Further, the attained results reveal that administration of CA4 leads to biochemical changes that are expressed as extracellular metabolome changes measurable in cell culture medium. As the current protocol did not induce cell lysis via addition of buffers or through other means, the measured differences can thus be assumed to be directly related to the efflux of molecules from cells towards the medium, presenting a true representation of extracellular metabolome changes.

As the SPME sorbent phases used in this work do not extract proteins, and in particular enzymes, SPME thus enables the extraction of short-lived small molecules. This feature of SPME retains the integrity of the available biological information, providing a true ‘snapshot’ of the system under study^14^. In the present work, a number of discriminant molecules able to distinguish between treated and control cells were putatively identified by comparison of accurate mass with the available databases e.g. Human Metabolome Database or ChemSpider database. Taking into account the main objective of the investigation, which was the demonstration of the applicability of the method to cell line study rather than in-depth biological interpretation of the data, the metabolites selected for detailed description were only those for which the p-value was below 0.05, fold-change was at least 2 and the biological role in cell line studies or and/or cancer studies has been reported previously. The current work also took into consideration the possibility that administration of CA4 may not only affect the metabolic pathways of cells, but also decrease the number of cells growing in media, which would be expressed in group separations in the principal component analysis (PCA) plot. This would consequently lead to selection of discriminating compounds based on different levels originating from mentioned decrease of cells numbers. As such, an additional control was applied to verify whether the observed metabolite changes were due to biochemical pathway alterations, or due to the decreased number of cells. The control group in this case was consisted of samples comprised of a reduced cell inoculum (1000 cells per well). In order to assess the impact of decreased cell growth, these samples were then compared and contrasted with samples treated with IC50 CA4 (2000 cells per well). Further verification regarding the number of cells in treated and control samples was carried out via SRB staining, which showed there were no statistical differences in the number of cells present in treated and untreated samples, with inhibition of growth measured at 17.5% ± 2.9 and 20.3% ± 1.8, respectively. Such a finding confirms that the different levels of metabolites documented in the metabolomics study are owed to alterations in cell metabolism, and not from differences in cell numbers.

**Figure 6:**
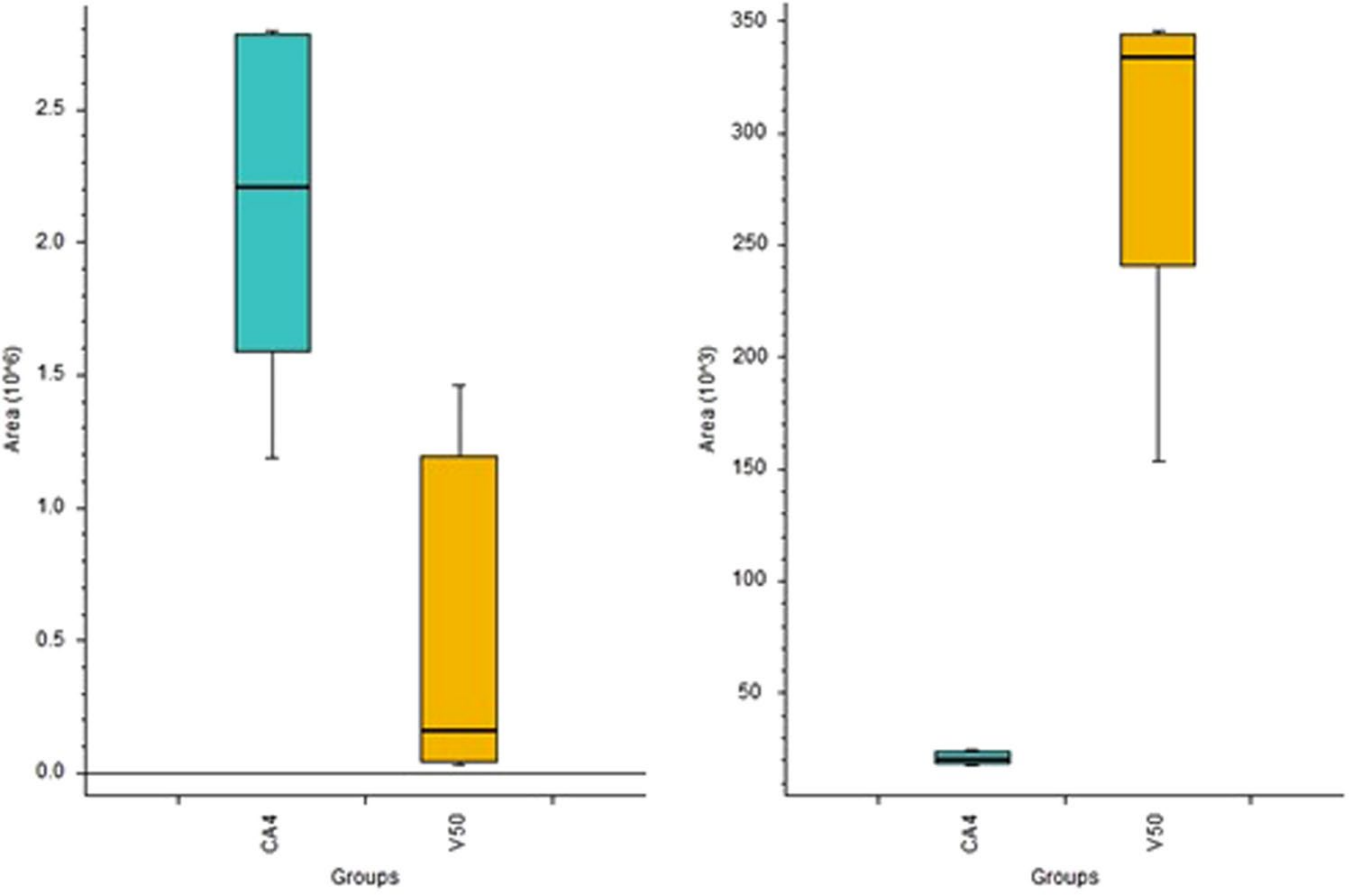
Box Whisker charts representing levels of neurine (left panel) and L-arginine (right panel). CA4 represents samples treated with combretastatin A4, and V50 represents control cells with an initial number of seeded cells equal to 1000.

Figure 6. provides a comparison of results for cells treated with CA4 (inoculum of 2000 cells were seeded) and the non-treated control, which had an inoculum of 1000 seeded cells. Peak areas reported for extracted ion chromatograms represent levels of putatively identified neurine and L-arginine. Neurine, a metabolite of choline degradation, with the ability to reduce proliferation of the neuroblastoma cell line^24^. Moreover, previous work has revealed that choline, by its conversion to phosphorylcholine, is involved in oncogenesis by enhancing cell metabolism and proliferation rates. Elevated levels of the kinase responsible for this transformation have been previously correlated with low survival prognoses for patients suffering from NSCLC. The *in vitro* portion of the same research, which employed different lung tumor cell lines as models, yielded results corresponding to the overexpression of the abovementioned kinase, thus providing evidence for the role of choline in carcinogenesis^25^. L-arginine, in turn, is an amino acid involved in multiple biochemical pathways, including central carbon metabolism in cancer^26^. Arginine was also present in cell culture medium, with lower levels found in CA4-treated samples, suggesting enhanced metabolism of A549 cells.

**Figure 7:**
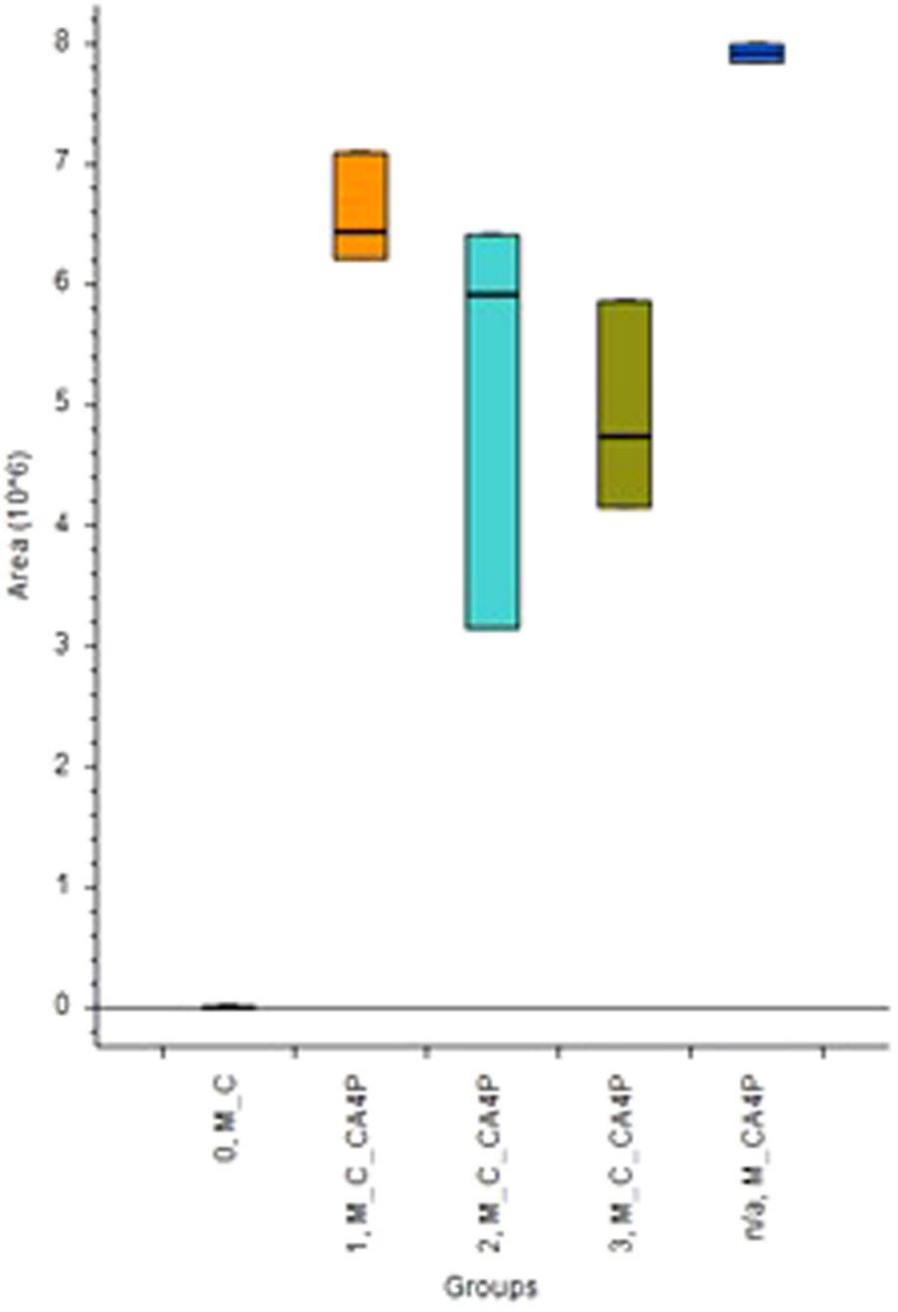
Diagram represents Box Whisker chart for CA4, where 0_M_C are the control cells unexposed to CA4P; M_C_CA4P are the cells exposed to CA4P at different time points T1-1, M_C_CA4P; T2-2, M_C_CA4P; T3-3, M_C_CA4P M_CA4P-medium control without cells spiked with CA4P.

**Figure 8:**
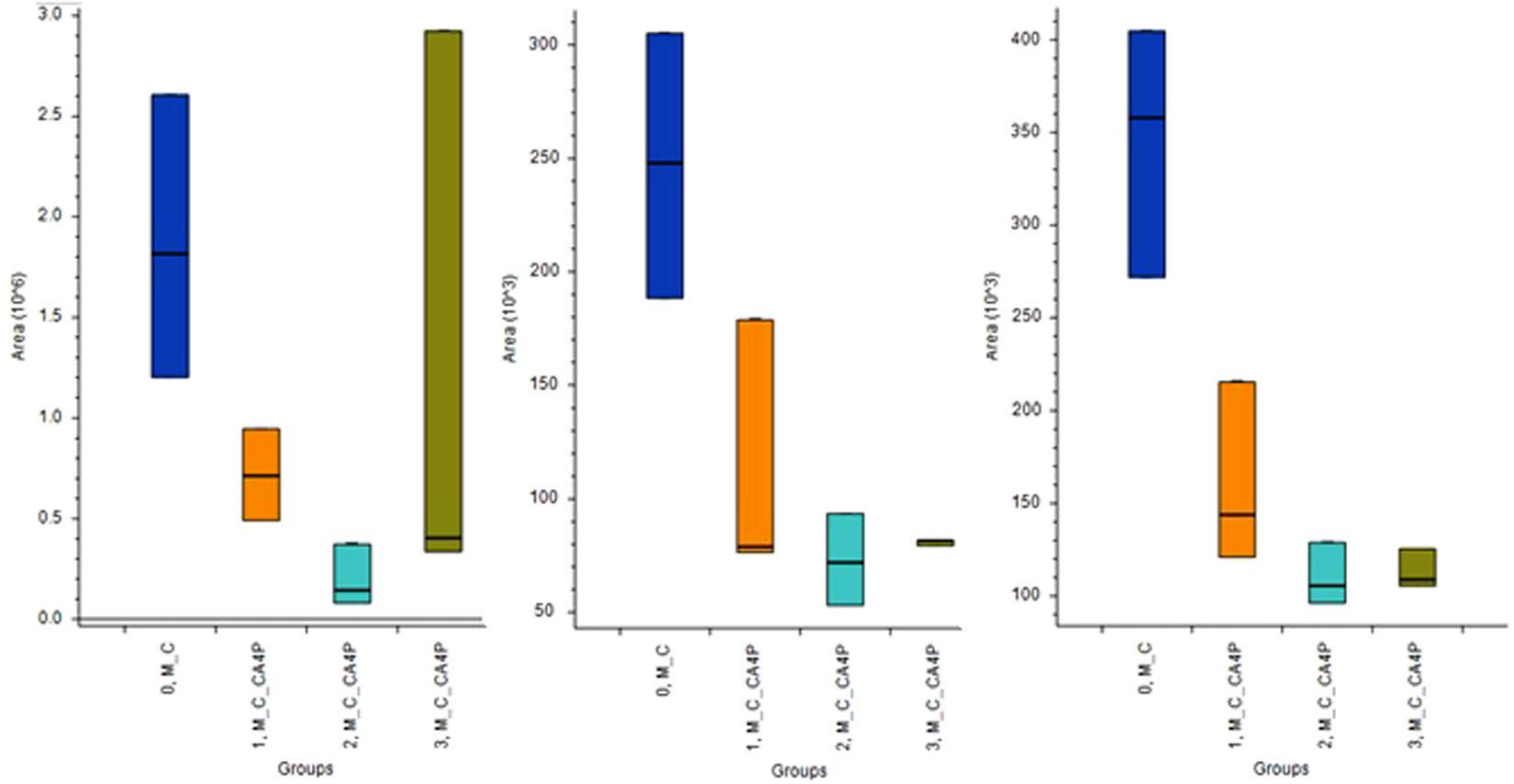
Box Whisker charts for palmitamide (left), lumichrome (middle) and L-homophenylalanine (right), where 0_M_C are control cells unexposed to CA4P; M_C_CA4P are cells exposed to CA4P at different time points 1, M_C_CA4P; 2, M_C_CA4P; 3, M_C_CA4P M_CA4P-medium control without cells spiked with CA4P.

Employment of protocol 2 and a time course analysis, the active form of the drug (CA4) was identified in extracts after administration of prodrug CA4P to the cell medium (Fig. 7.). This is due to the presence of phosphatases in the cell medium, which was enriched with fetal bovine albumin. As can be seen, CA4 was also found in samples containing medium without cells (Fig. 7. M_CA4P), as well as cells treated with CA4P (Fig. 7. 1, 2 and 4 M_C_CA4P), but not in cells that were not treated with the prodrug (Fig. 7. M_CA4P). This finding is in good agreement with previous work, which pointed at the *in vivo* transformation of CA4P into CA4 with T_1/2_ below 30 min, as well as the presented hypothesis that the phosphate bond of prodrug can be cleaved by unspecific phosphatase^27^. Our results show that CA4P and CA4 can be used exchangeably in *in vitro* cell-based assays. Moreover, time course analysis revealed that the concentration of CA4 in cell medium gradually decreased over time, a phenomenon that can be attributed to the intake of the drug by cells. The untargeted metabolomics approach revealed time-dependent changes in the levels of three metabolites: palmitamide, lumichrome, and L-homophenylalanine (Fig. 8.). Previous works have shown that decreased levels of palmitamide correlate with colon cancer cell line resistance following administration of 5-FU^28^. Down regulation of palmitamide has also been found to correlate with the survival rates of patients afflicted by colorectal cancer^29^. Lumichrome is associated with riboflavin (vitamin B2) metabolism^26^, riboflavin being a component of cell culture medium. Thus, our results indicate that riboflavin metabolism was also influenced after administration of CA4. L-homophenylalanine, in turn, plays a role as an intermediate substrate in various metabolic pathways; as such, its involvement in CA4 activity with respect to A549 cells must be further explored. Succinctly, the results obtained in the current study confirm that SPME is a suitable tool for monitoring of absorption of targeted drugs, as well as for metabolomics analysis of the studied cell line models.

## Conclusions

Solid phase microextraction is presented in the current work as a suitable tool for high throughput tracking of metabolic changes in *in vitro* systems, either as part of one time-point investigations, or in time course studies, in which case SPME additionally offers the possibility of multiple analyses from the same samples due to its minimally-invasive nature, and negligible depletion capabilities. The different fiber lengths of the SPME devices used for the described protocols demonstrate the flexibility of the SPME method: the short coating enables direct sampling for time course analysis from low volumes in 96-well plates, while on the other hand, longer coatings can be employed in cases where multiple samplings are not needed, as is the case in time-course analyses, thus increasing the sensitivity of the method as well as the chances of finding metabolites present at very low concentrations. The current work corroborates that treatment of a non-small cell lung cancer cell line with combretastatin A4 and its prodrug, combretastatin A4 phosphate, results in alteration of cell metabolism. This is supported by the observed changed levels of endogenous compounds after administration of CA4 or CA4P. The *In vitro* cell-based platform is capable of metabolizing the prodrug of CA4, combretastatin A4 phosphate CA4P, to its active form. Moreover, the current work demonstrated that the intake of the drug by cells can be measured with SPME by means of temporal resolution. As employment of SPME does not disturb cell growth and only requires minimal sample consumption, SPME is thus shown to be compatible with routine cell culture protocols, as it enables the execution of other assays, such as SRB staining for cytotoxicity assessment, for instance, from the same sample, thus allowing for overall higher analysis precision as well as lower sample consumption.
